# Supplementary material for: TSST-1 promotes colonization of Staphylococcus aureus within the vaginal tract by activation of CD8+ T cells
Source: Infect Immun. 2025 Jan 22;93(2):e00439-24. doi: 10.1128/iai.00439-24 (PMC11834441; doi:10.1128/iai.00439-24)
Supplement: Supplemental material — Fig. S1 to S4; Tables S1 and S2. [file iai.00439-24-s0001.pdf]

SUPPLEMENTARY MATERIAL

TSST-1 promotes colonization of *Staphylococcus aureus* within the vaginal tract by activation of CD8$^+$ T cells

Karine Dufresne[1], Kait F. Al[1,2], Heather C. Craig[1], Charlotte E.M. Coleman[1], Katherine J. Kasper[1], Jeremy P. Burton[1,2], John K. McCormick[1,2]

[1] Department of Microbiology and Immunology, Schulich School of Medicine and Dentistry, University of Western Ontario, London (ON), Canada

[2] Canadian Centre for Human Microbiome and Probiotics Research, Lawson Health Research Institute, London, Ontario, Canada

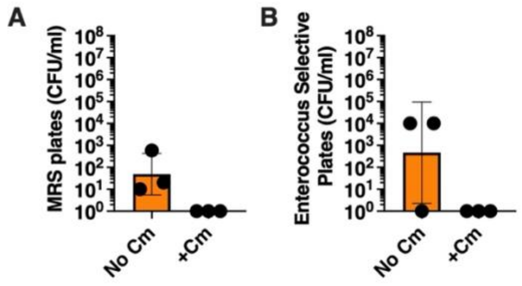

Figure S1. Confirmation of microbiota-depletion by chloramphenicol treatment. Presumptive lactobacilli on MRS plate (**A**) and enterococci on Enterococcus Selective Plates (**B**) from mice supplied with water either with or without chloramphenicol. No CFUs were recovered on either media when chloramphenicol was added to the drinking water. Each dot represents an individual mouse.

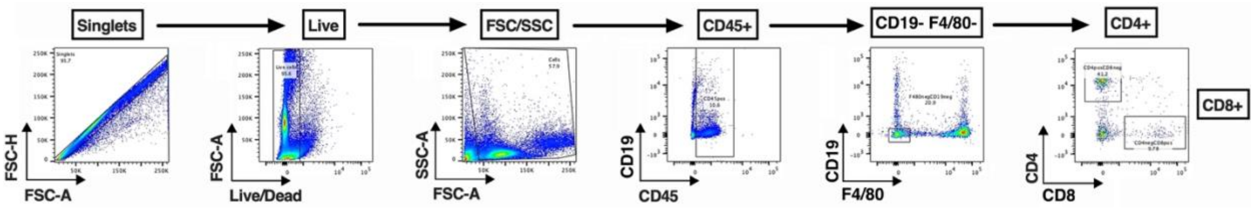

Figure S2. Strategy for gating in the flow cytometry experiment. Representative flow plots for gating strategy used to discriminate CD45^{+} and CD4^{+} and CD8^{+} cells. First, the single events were isolated and dead cells were excluded (negative for the viability dye) and then CD45-positive cells were gated, followed by gating on CD19- and F4/80- populations. From this population, cells were gated for either CD4-positive or CD8-positive cells. The analysis was applied to all samples. Each group had 3 biological replicates and were then plotted in graph in Figure 3A-C.

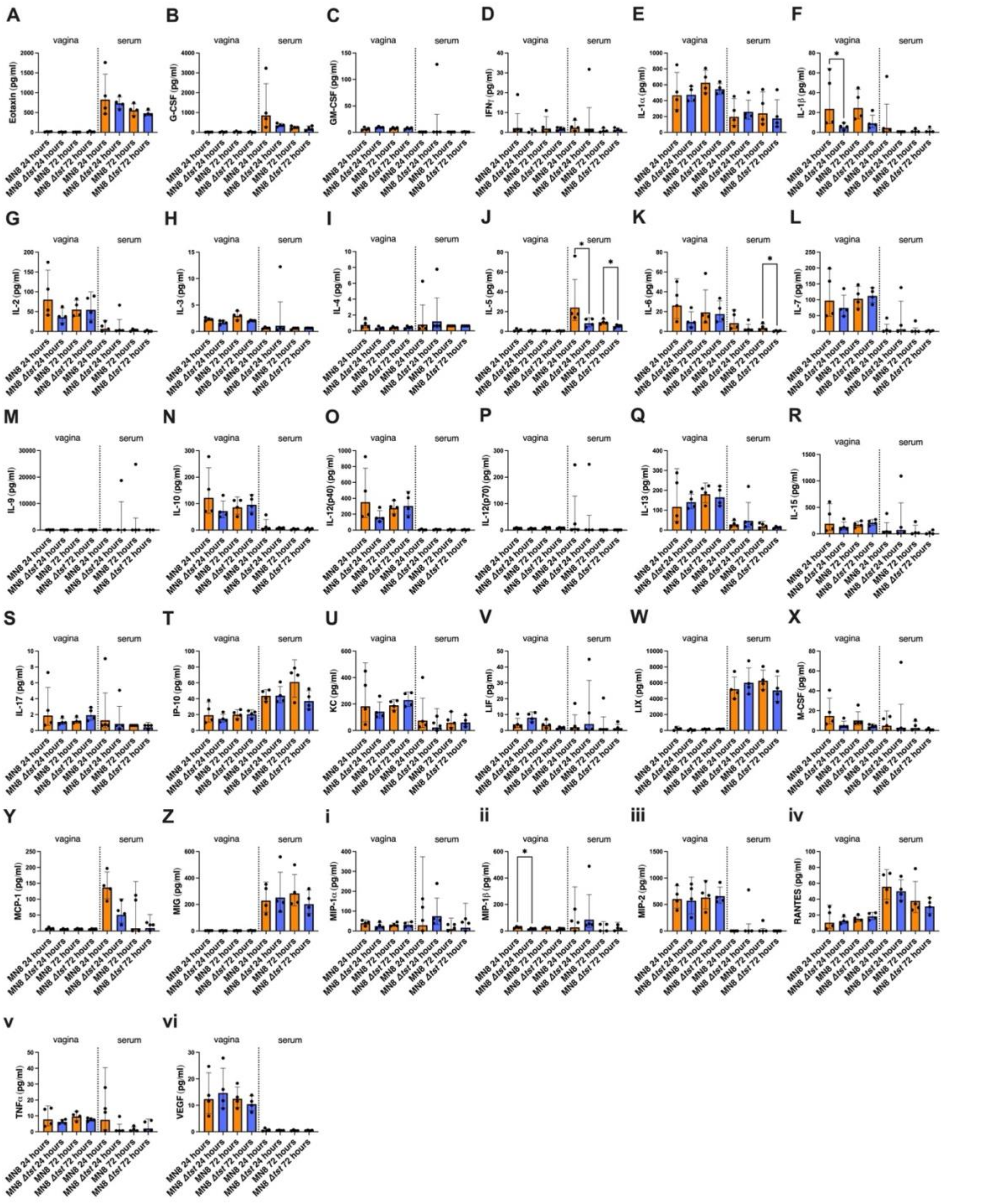

Figure S3. Quantitative cytokine response from BALB/c mice in vaginal homogenates and serums during staphylococcal infection. Mice were infected intravaginally with either 106 CFU/ml of *S. aureus* MN8 (orange bars) or MN8*Dtst* (blue bars). Mice were sacrificed 24- or 72 hours later and complete vaginal homogenates or serums were analyzed for multiple cytokines and chemokines [(A) Eotaxin; (B) G-CSF; (C) GM-CSF; (D) IFNg; (E) IL-1a; (F) IL-1b; (G) IL-2; (H) IL-3; (I) IL-4; (J) IL-5; (K) IL-6; (L) IL-7; (M) IL-9; (N) IL-10; (O) IL12(p40); (P) IL-12(p70); (Q) IL-13; (R) IL-15; (S) IL-17; (T) IP-10; (U) KC; (V) LIF; (W) LIX; (X) M-CSF; (Y) MCP-1; (Z) MIG; (i) MIP-1a; (ii) MIP-1b; (iii) MIP-2; (iv) RANTES; (v) TNFa; (vi) VEGF]. Data represent the geometric mean ± SD of vaginal or serum cytokine/chemokine concentration (pg mL−1) (n ≥ 4 mice per group).

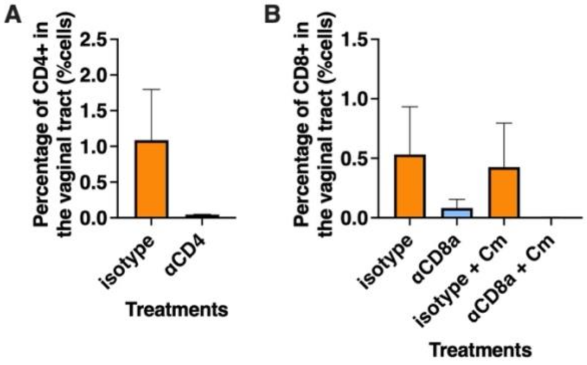

Figure S4. Flow cytometric analysis of vaginal cell populations post T-cell depletion (n = 2 per group). Percentage of (A) $CD4^+$ and (B) $CD8^+$ cells to $CD45^+$ T lymphocyte populations in the isotype and treatment groups. Data are shown as mean ± SEM.

Table S1. Sample read counts

Sample	Total read count	Retained in downstream analyses
S72A_1	300	No
S72A_2	7630	Yes
S72A_3	351	No
S72A_4	18455	Yes
S72A_5	356	No
S72A_6	14	No
S72A_7	425318	Yes
S72A_8	645	No
TSST1_1	3433	Yes
TSST1_2	6140	Yes
TSST1_3	554626	Yes
TSST1_4	1598	Yes
TSST1_5	703	No
TSST1_6	135699	Yes
TSST1_7	219529	Yes
TSST1_8	884	No
**Average**	**85980**	

Table S2. Ten most differential taxa between animal groups. Negative effect size values are taxa relatively enriched in S72A, positive effect size values are taxa relatively enriched in TSST1. CLR = centered log-ratio transformed values. Complete sequences reads have been deposited in BioProject ID PRJNA1117910.

SV	Taxonomy (Phylum;Class;Genus)	Median CLR value in S72A	Median CLR value in TSST1	Effect Size
**SV_47**	Bacillota; Clostridia; *Lachnospiraceae* (NK4A136)	5.22968123	-0.8597584	-1.2042668
**SV_37**	Bacillota; Clostridia; *Lachnospiraceae* (NK4A136)	1.45463548	-0.9938375	-0.6147517
**SV_82**	Bacillota; Clostridia; *Lachnospiraceae* (NK4A136)	1.52978144	-0.9360268	-0.5963797
**SV_23**	Bacillota; Clostridia; unclassified *Lachnospiraceae*	1.6709751	-0.9539195	-0.5860112
**SV_92**	Bacillota; Clostridia; *Lachnospiraceae* (NK4A136)	1.67133591	-1.0780277	-0.5696867
**SV_114**	Bacillota; Clostridia; unclassified *Lachnospiraceae*	1.78130369	-0.753003	-0.5621407
**SV_65**	Bacillota; Clostridia; *Lachnospiraceae* (UCG-006)	1.72160682	-0.9996819	-0.5613534
**SV_64**	Bacillota; Clostridia; *Lachnospiraceae* (NK4A136)	1.71061117	-1.0880001	-0.5570048
**SV_71**	Bacillota; Clostridia; *Dorea*	1.2965907	-0.9497423	-0.5565569
**SV_81**	Actinomycetota; Actinomycetes; *Lawsonella*	1.6471348	-0.8975239	-0.5562579
**SV_154**	Pseudomonadota; Betaproteobacteria; *Aquabacterium*	-1.8607425	3.36612894	0.68806882
**SV_3**	Pseudomonadota; Gammaproteobacteria; *Proteus*	1.62622062	8.01405487	0.69207961
**SV_21**	Bacillota; Bacilli; *Ligilactobacillus*	4.05439365	8.15139279	0.7264859
**SV_8**	Pseudomonadota; Alphaproteobacteria; *Afipia*	5.22744687	7.46638074	0.72695204
**SV_17**	Pseudomonadota; Gammaproteobacteria; *Proteus*	-2.3679833	1.19626484	0.73304675
**SV_70**	Bacillota; Bacilli; *Enterococcus*	-2.6808654	1.01918525	0.74909067
**SV_16**	Bacillota; Bacilli; *Enterococcus*	-2.0073549	3.49692934	0.76279031
**SV_4**	Pseudomonadota; Gammaproteobacteria; *Proteus*	0.11181648	7.27293703	0.78216589
**SV_12**	Bacillota; Bacilli; *Enterococcus*	-2.3124189	5.16044384	0.82092718
**SV_2**	Pseudomonadota; Gammaproteobacteria; *Proteus*	-2.1178604	10.2800895	1.26206171
